# Predicting Odor Perceptual Similarity from Odor Structure

**DOI:** 10.1371/journal.pcbi.1003184

**Published:** 2013-09-12

**Authors:** Kobi Snitz, Adi Yablonka, Tali Weiss, Idan Frumin, Rehan M. Khan, Noam Sobel

**Affiliations:** Department of Neurobiology, Weizmann Institute of Science, Rehovot, Israel; University College London, United Kingdom

## Abstract

To understand the brain mechanisms of olfaction we must understand the rules that govern the link between odorant structure and odorant perception. Natural odors are in fact mixtures made of many molecules, and there is currently no method to look at the molecular structure of such odorant-mixtures and predict their smell. In three separate experiments, we asked 139 subjects to rate the pairwise perceptual similarity of 64 odorant-mixtures ranging in size from 4 to 43 mono-molecular components. We then tested alternative models to link odorant-mixture structure to odorant-mixture perceptual similarity. Whereas a model that considered each mono-molecular component of a mixture separately provided a poor prediction of mixture similarity, a model that represented the mixture as a single structural vector provided consistent correlations between predicted and actual perceptual similarity (r≥0.49, p<0.001). An optimized version of this model yielded a correlation of r = 0.85 (p<0.001) between predicted and actual mixture similarity. In other words, we developed an algorithm that can look at the molecular structure of two novel odorant-mixtures, and predict their ensuing perceptual similarity. That this goal was attained using a model that considers the mixtures as a single vector is consistent with a synthetic rather than analytical brain processing mechanism in olfaction.

## Author Summary

One hundred years ago, Alexander Graham Bell asked: “Can you measure the difference between one kind of smell and another? It is very obvious that we have very many different kinds of smells, all the way from the odor of violets and roses up to asafetida. But until you can measure their likenesses and differences you can have no science of odor.” Here we developed a computational framework and algorithm that looks at the molecular structure of two odors and predicts their ensuing perceptual similarity. Importantly, the algorithm works for odors that are each composed of a mixture containing tens of different molecules, much like natural smells. The algorithm that worked best was one that treats the odor-mixture as a single value, rather than a bunch of values reflecting each of its components. This is consistent with the growing view of how the mammalian brain treats odors: synthesizing a singular odor percept rather than analytically extracting individual odorant features from the odor-mixture. In conclusion, our algorithm's performance may contribute to the practice of the science of odor, and our algorithm's nature may contribute to the understanding of brain mechanisms of smell.

## Introduction

One hundred years ago, Alexander Graham Bell asked: “Can you measure the difference between one kind of smell and another. It is very obvious that we have very many different kinds of smells, all the way from the odor of violets and roses up to asafetida. But until you can measure their likenesses and differences you can have no science of odor.” [1]. Although the challenge posed by Bell has been widely recognized in olfaction research [2], [3], the field has yet to gravitate to an agreed upon system for odor measurement.

Early investigations into quantification of odor revolved around an effort to identify odor primaries, similar to the notion of primary colors in vision [4]. A major tool in this effort was the quantification of specific anosmias [5]. Although specific anosmia remains a powerful tool for linking odor perception to olfactory neurobiology [6], [7], this path did not generate a general method to quantify olfactory perception. A conceptually similar approach was an effort to identify specific odorant molecular features that drove specific olfactory perceptual notes. This approach, referred to as structure-odor-relationships or SOR [8], identified many specific rules linking structure to odor (e.g., what structure provides a “woody” note), but failed to produce a general framework for measuring smell.

An alternative path to measuring smell was to identify general perceptual primaries rather than individual odorant primaries [9]–[12]. This approach, consisting of applying statistical dimensionality reduction to many perceptual descriptors applied to many odorants, repeatedly identified *odorant pleasantness*, namely an axis ranging from very unpleasant to very pleasant, as the primary dimension in human olfactory perception [13]–[18]. Initial efforts to link such perceptual axes to odorant structural axes saw only limited success because of the limited scope of physicochemical features one could easily obtain for a given molecule [19]. However, the recent advent of software that provides thousands of physicochemical descriptors for any molecule (e.g., Dragon software, Talete, Milan, Italy) now allowed application of similar dimensionality reduction to odorant structure as well. This process revealed odorant structural dimensions that were modestly but significantly predictive of odorant perception [17] and odorant-induced neural activity across species [20]–[24].

Although the above studies combine to generate an initial form of olfactory metrics, they all apply to mono-molecular odorants alone. The real olfactory world, however, is not made of mono-molecules, but rather of complex olfactory multi-molecular mixtures. For example, roasted coffee [25], red wine [26], or rose [27], each contain hundreds of different mono-molecular species, many of them volatile. Thus, a useful metric for smell must apply to such odorant-mixtures. Although an ultimate metric would predict exactly how such mixtures smell in verbal descriptor terms, an initial interim goal is to predict their perceptual similarity. With this in mind, we collected perceptual similarity estimates from a large group of subjects rating a large group of odorant-mixtures of known components. We then tested alternative models linking odorant-mixture structure to odorant-mixture perceptual similarity, and identified a model and algorithm that provided a meaningful predictive framework. Using this algorithm we can now look at two novel mono-molecular odorants, or multi-component odorant-mixtures, and predict a significant portion of their ensuing perceptual similarity.

## Results

### Selecting components for odorant-mixtures

**Figure 1:**
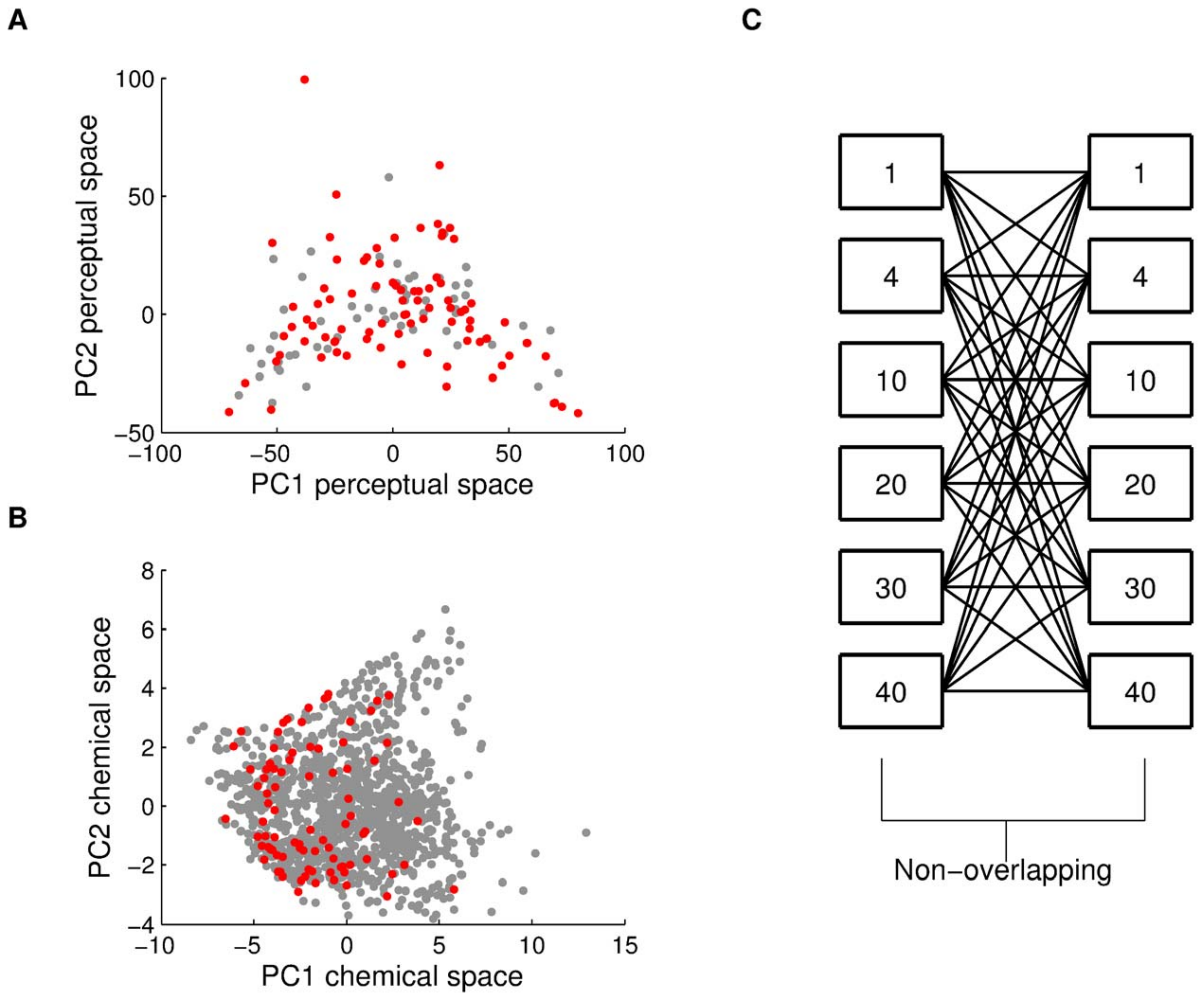
Odorant selection and comparison. The odorants we used are plotted in red, presented within: (**A**) Perceptual space: 138 odorants commonly used in olfaction research, projected onto a two-dimensional space of PC1 (30.8% of the variance) and PC2 (12% of the variance) of perception. (**B**) Physicochemical space: 1358 odorants commonly modeled in olfaction research projected onto a two-dimensional space made of PC1 (37.7% of the variance) and PC2 (12.5% of the variance) of structure. (**C**) A schematic reflecting mixture comparisons in Dataset #1 in Table S2. Each mixture was compared to all other mixtures with zero overlap in component identity, and to itself. Note that this schematic reflects one quarter of the data, as we had eight versions of each mixture size.

Odorants can generally be described by a large number of perceptual or structural descriptors. Dravnieks' atlas of odor character profiles includes 138 mono-molecules, each described by 146 verbal descriptors of perception. We call this the ‘perceptual odor space’. Odorants can also be described by a large set of structural and physicochemical descriptors. We selected 1358 odorants commonly used in olfaction research, and obtained 1433 such descriptors using Dragon software (v. 5.4, Talete, Milan, Italy) (note that Dragon provides 1664 descriptors, but 231 descriptors were without values for the molecules we modeled). Since the different descriptors measure properties on differing scales we normalized the Dragon data so that the values of each descriptor ranged between 0 and 1. That is, for each descriptor *d* we have a set of 1358 values *ld* (barring missing values). Each values *v* in the list *ld* is normalized to the value *vn* by the equation
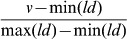
(1)We call this normalized data the ‘physicochemical odor space’ (Table S1 contains the odorants we modeled and their descriptor values). To form odorant-mixtures, we obtained 86 mono-molecular odorants that were well-distributed in both perceptual (Figure 1A) and physicochemical (Figure 1B) stimulus space (Dataset #1 in Table S2). We then diluted each of these odorants separately to a point of about equal perceived intensity as estimated by an independent group of 24 subjects, and prepared various odorant mixtures containing different numbers of such equal-intensity odorant components. Importantly, to prevent formation of novel compounds, odorant mixtures were not mixed in the liquid phase, but rather each component was dripped onto a common absorbing pad in a sniff-jar, such that their vapors alone mixed in the jar headspace (the integrity of this method was later verified in Dataset #2 in Table S2 using gas-chromatography mass-spectrometry (GCMS), see Methods). We prepared several different versions for each mixture size containing 1, 4, 10, 15, 20, 30, 40 or 43 components, such that half of the versions were well-spread in perceptual space, and half of the versions were well-spread in physicochemical space.

We conducted pairwise similarity tests, using a visual analogue scale (VAS) (see Methods), of 191 mixture pairs, in 48 subjects (24 women, average of 14 subjects per comparison). Each target mixture (1, 4, 10, 15, 20, 30, 40 or 43 components) was compared to all other mixtures (1, 4, 10, 15, 20, 30, 40 or 43 components), and as a control, to itself. Other than comparisons of a mixture to itself (44 comparisons), all comparisons were non-overlapping (147 comparisons), i.e. each pair of mixtures under comparison shared no components in common (Figure 1C) (Table S2 contains all the similarity estimates for the three datasets used in this study).

### The pairwise distance model for odorant-mixture similarity

**Figure 2:**
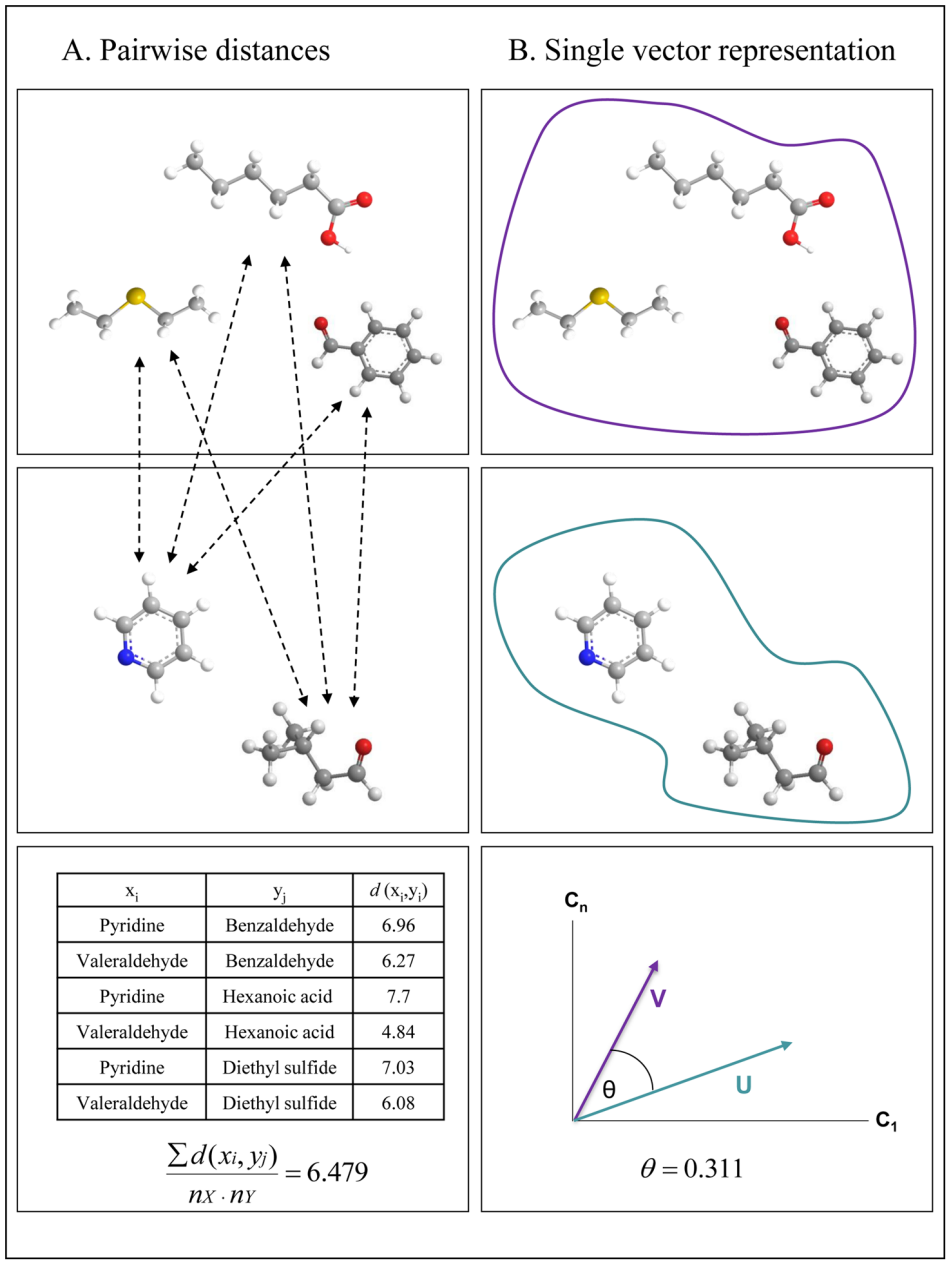
Modeling odorant mixtures as singular objects rather than component amalgamations. The top panels represent one mixture (Y) made of 3 mono-molecular components and the bottom panels represent a different mixture (X) made of 2 mono-molecular components. The distance between X and Y can be calculated as (**A**) The mean of all pairwise distances between all the components of X and Y. (**B**) Alternatively, one can represent both X and Y as single vectors reflecting the sum of their components, and define the distance between them as the angle between these two vectors within a physicochemical space of n dimensions.

**Figure 3:**
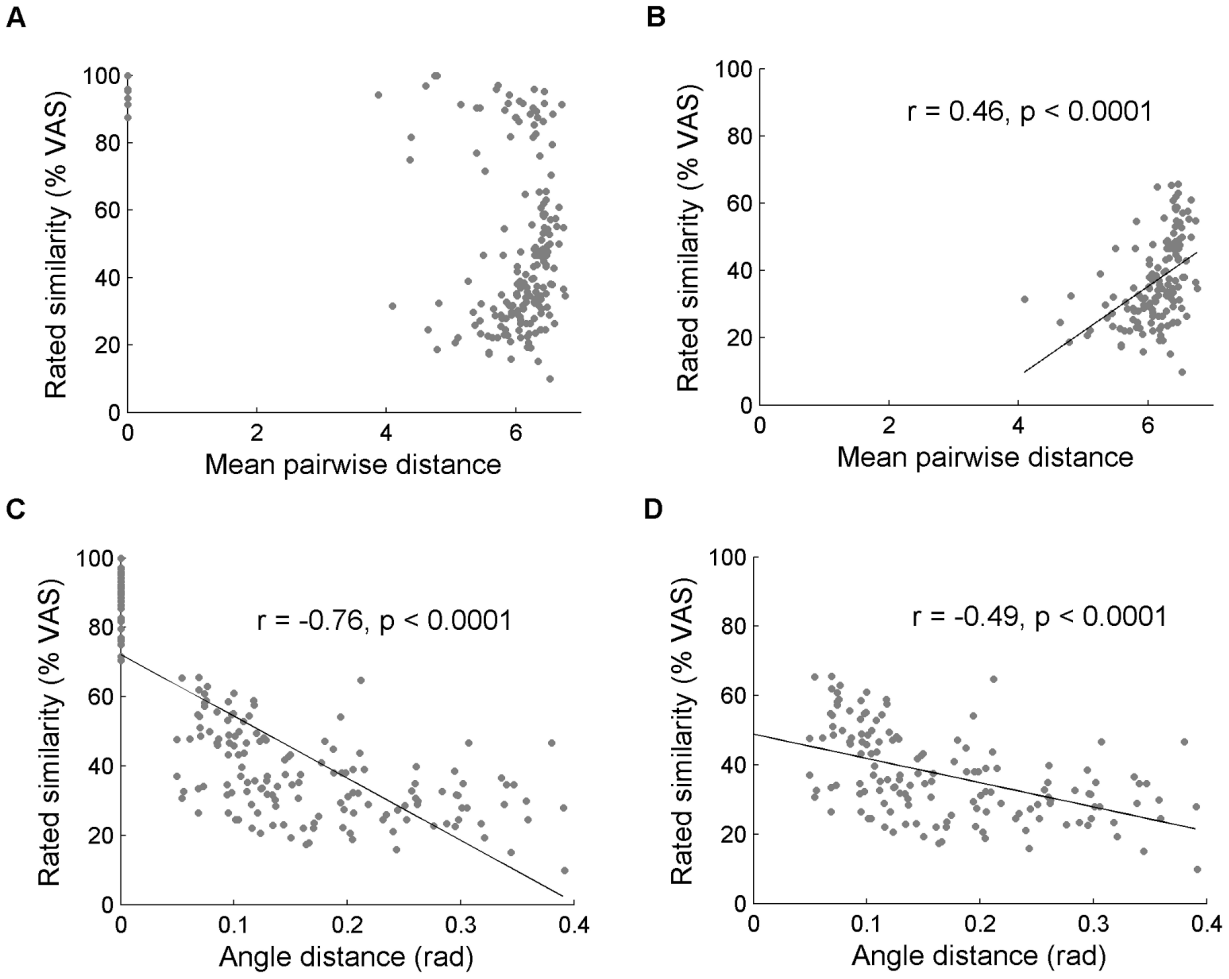
Performance of the pairwise distance and angle distance models. Each dot reflects a comparison between two odorant mixtures. (**A**) The pairwise distance model was not predictive of mixture similarity. (**B**) Removing comparisons of a mixture to itself, the pairwise distance model implies a non-logical point from which increases in structural similarity drive decreases in perceived similarity. (**C**) The angle distance model provides a strong prediction of perceived similarity. (**D**) The angle distance model continues to provide logical results after removing comparisons of mixtures to themselves.

One simple model for predicting the perceptual difference between mixtures is to measure all pairwise Euclidean physicochemical distances between all individual mixture components, and then average them. This approach treats each mixture component individually (Figure 2A). To test this model, we obtained the 1433 physicochemical descriptors for each of the 86 mono-molecular components we used. We found that the mean pairwise Euclidean distance over all the descriptors of all mono-molecular components comprising any two mixtures was a poor predictor of perceptual similarity between the two mixtures. The relationship between pairwise-distance and perceived similarity did not fit any simple model, linear or other (Figure 3A). Moreover, the distribution of this relationship was clearly skewed by the similarity ratings given to the comparisons of a mixture to itself, yet eliminating these comparisons revealed a significant correlation in the opposite direction (r = 0.46, p<0.0001) (Figure 3B). In other words, this model implied that odor-mixtures identical in structure will be the furthest apart in perceptual similarity. Given this clear failing-point of the model, we set out to investigate an alternative model.

### The angle distance model for odorant-mixture similarity

An alternative model is to consider the mixture as a whole rather than a set of constituents (Figure 2B). To test this, we used the same 1433 physicochemical descriptors for each mono-molecular mixture component, but this time we created a single vector representing the whole mixture by summing the vectors of its components. To eliminate the effect of the number of components in a mixture on the size of the mixture vector, we divided it by its norm. Thus, each mixture was now represented by a vector made of 1433 descriptors. We then defined the distance between the vector of mixture U and the vector of mixture V, as the angle between the two vectors, given by:
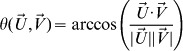
(2)where U·V is the dot product between the vectors, and |U|,|V| are the norms of the vectors. We found that this angle distance was strongly predictive of perceived mixture similarity (r = −0.76, p<0.0001) (Figure 3C). Omitting comparisons of mixtures to themselves resulted in a correlation of r = −0.49, p<0.0001 (Figure 3D). Unlike the pairwise distance model, this model did not predict that physically identical mixtures would in fact smell dissimilar. Therefore, we set out to optimize this model.

### Optimizing the angle distance model

In order to optimize the model, we first set out to collect an independent dataset (Dataset #2 in Table S2). To address the possibility that the performance of our model was somehow influenced by the nature of our mixtures, whose components were selected to span olfactory space, the components for Dataset #2 in Table S2 mixtures were selected randomly. We randomly selected 43 molecules out of the 86 equated-intensity molecules, and made 13 mixtures of 4–10 randomly selected components. Thus, unlike in Dataset #1 in Table S2, and more like odors in the real world, here there was some overlap in components across mixtures. Twenty-four subjects (13 women) conducted pairwise similarity tests of all 91 possible pairs plus 4 comparisons of identical mixtures for a total of 95 comparisons (each such comparison was repeated twice). Subjects conducted the similarity tests within four sessions on four consecutive days (∼48 comparisons per day). Comparisons were counter-balanced for order.

### Model optimization: Selecting chemical descriptors through simulation

We set out to extract the most relevant chemical descriptors for predicting perceptual similarity using the angle distance model. In order to do so, we needed to compare the quality of predictions based on different combinations of descriptors. However, because our data includes 1433 different descriptors, it was impossible to compare all possible selections of descriptors in order to pick the best performing selection (2^1433^ possibilities). With this in mind, we first set out to model the total number of descriptors our model would rely on.

### Step 1: Selecting the number of descriptors

**Figure 4:**
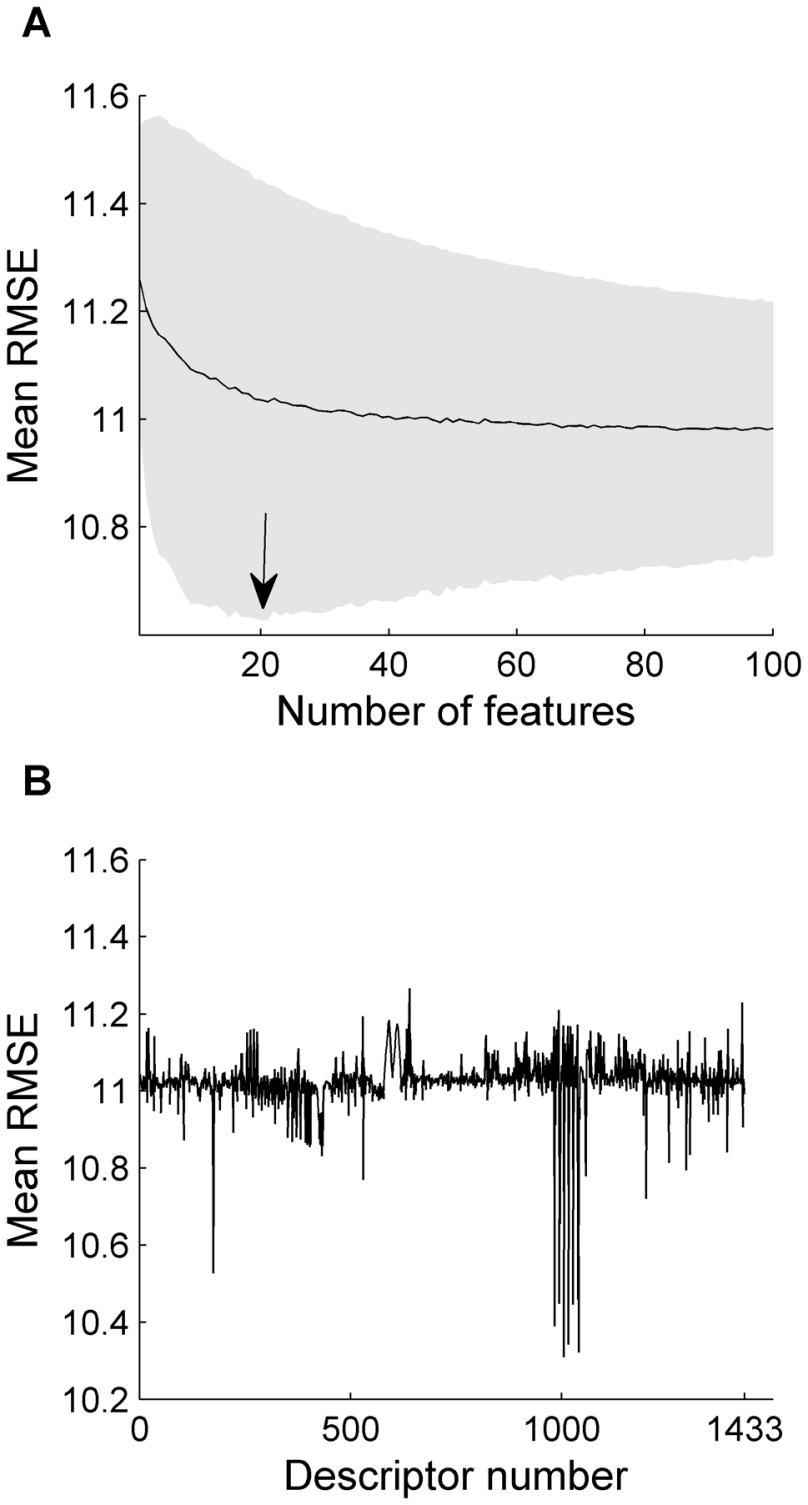
Optimizing the angle distance model. (**A**) Mean RMSE for varying number of features. Plotted in grey are the standard error values for each number of features. The lowest value was obtained at about 20. (**B**) Mean RMSE per descriptor. For each of the 1433 descriptors, the mean RMSE was calculated between the similarity ratings of mixture pairs and the angle distance model based on 2,000 selections of 25 random descriptors, one of which is the fixed descriptor in question. A score was given to each descriptor based on this mean RMSE for the next step.

The first step in our optimizing method was to decide on the number of features (descriptors) we were going to look for. To do this we used a random half of Dataset #2 in Table S2 as a training-set (47 comparisons) and ran a simulation on it. In the simulation we ran through each number of features from 1 to 100. For each number of features *n* we selected 20,000 random samples of descriptors sized *n* and calculated the root mean square error (RMSE) for the prediction on the training set comparisons based on these descriptors. For each *n* we then calculated the mean RMSE and the standard deviation and plotted the result (Figure 4A). At *n* = 20 the value of the mean RMSE minus the standard deviation was the lowest (Figure 4A, the trend continues to increase for *n*>100). This told us that at around 20 descriptors, we should expect the selections that would produce the lowest RMSE. Since our feature selection method includes the possibility of selecting a feature twice we searched for slightly larger size sets of features so that at the end of the process we would have about 20 descriptors.

### Step 2: Evaluating individual descriptors

Although we could have compared the performance of a selection of descriptors, we wanted to estimate the relevance of individual descriptors. If we selected 25 descriptors at random out of the 1433 and based our predictive model on them, we were likely to obtain a prediction that correlated to an RMSE of about 11 (Figure 4A). However in order to optimize our model we wanted to distinguish those descriptors which give rise to more accurate predictions from those that do not. In order to evaluate a descriptor *d* in terms of how much it contributes to accurate predictions we ran a simulation for each descriptor. In the simulation for descriptor *d* we tested the predictive performance of a large number of randomly selected sets of descriptors to which we added descriptor *d*. We used 2000 random selections of 25 descriptors together with *d* and tested their predictive performance on the same training and testing set from before. For each selection we calculated the RMSE, and then calculated the mean RMSE across the 2000 selections. This mean is the number assigned to descriptor *d* (Figure 4B), giving us an indication of how relevant the descriptor *d* is to making similarity predictions: the lower the mean RMSE, the more relevant *d* is. Figure 4B is a plot of these averages calculated for each one of the 1433 descriptors. As apparent in the figure, for most descriptors the average performance for random selections that include them is about the same. However, some descriptors stand out.

### Step 3: Searching for the best selection of descriptors

**Figure 5:**
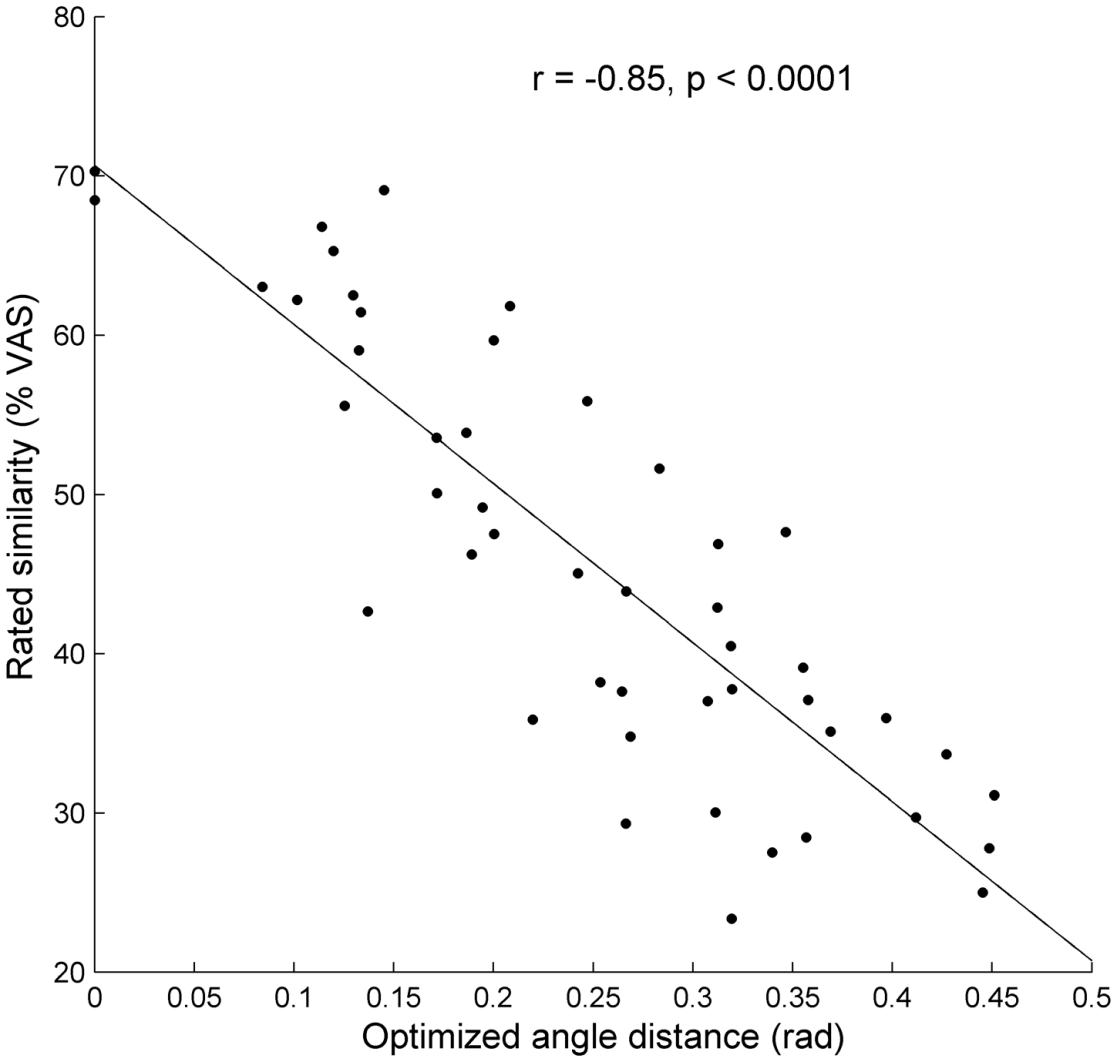
Performance of the optimized angle distance model. Each dot represents a comparison between two mixtures. The optimized model provided a strong prediction of mixture perceptual similarity from mixture structure alone.

The next step in our descriptor selection process was a second simulation where we selected 4000 samples of 25 descriptor sets based on the performance of the individual descriptors in the second step of the selection process. We gave each of our descriptors a non-negative score based on its mean RMSE calculated in the first part of the process. The score was calculated as
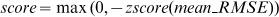
(3)so that only descriptors with an RMSE value lower than the average RMSE value (i.e. good-performing descriptors) were associated with a score greater than zero. Then we proceeded to select random samples according to the scores we just calculated. That is, in the third step of the process, those descriptors that performed better in the second step were more likely to be included in the (semi) random sample. Using this method we selected 4000 samples of 25 descriptors and picked the ones that performed best, i.e. the selection that produced the lowest RMSE in the training set predictions. We removed repeated descriptors from our best performing selection of 25 descriptors and obtained a selection of 21 descriptors that performed even better (Table 1). The performance of the descriptors selected according to this two-step training process was tested on the testing set and the resultant correlation between predicted odorant-mixture similarity and actual odorant-mixture similarity was RMSE = 6.98, r = −0.85, p<0.001 (Figure 5). Whereas the above random selection of descriptors may give rise to different descriptor subsets in recurring simulations, a deterministic selection of descriptors did not generate better results (Text S1 Section 1).

**Table 1:** List of 21 descriptors for optimized mixture similarity prediction.

No.	Index out of 1433 descriptors	Abbreviation	Description
1	19	nCIR	Number of circuits (constitutional descriptors).
2	44	ZM1	First Zagreb index M1 (topological descriptors).
3	51	GNar	Narumi geometric topological index (topological descriptors).
4	96	S1K	1-path Kier alpha-modified shape index (topological descriptors).
5	175	piPC08	Molecular multiple path count of order 08 (walk and path counts).
6	289	MATS1v	Moran autocorrelation – lag 1/weighted by atomic van der Waals volumes (2D autocorrelations).
7	295	MATS7v	Moran autocorrelation – lag 7/weighted by atomic van der Waals volumes (2D autocorrelations).
8	321	GATS1v	Geary autocorrelation – lag 1/weighted by atomic van der Waals volumes (2D autocorrelations).
9	351	EEig05x	Eigenvalue 05 from edge adj. matrix weighted by edge degrees (edge adjacency indices).
10	407	ESpm02x	Spectral moment 02 from edge adj. matrix weighted by edge degrees (edge adjacency indices).
11	423	ESpm03d	Spectral moment 03 from edge adj. matrix weighted by dipole moments (edge adjacency indices).
12	430	ESpm10d	Spectral moment 10 from edge adj. matrix weighted by dipole moments (edge adjacency indices).
13	433	ESpm13d	Spectral moment 13 from edge adj. matrix weighted by dipole moments (edge adjacency indices).
14	477	BELv3	Lowest eigenvalue n. 3 of Burden matrix/weighted by atomic van der Waals volumes (Burden eigenvalues).
15	733	RDF035v	Radial Distribution Function – 3.5/weighted by atomic van der Waals volumes (RDF descriptors).
16	994	G1m	1^st^ component symmetry directional WHIM index/weighted by atomic masses (WHIM descriptors).
17	1005	G1v	1^st^ component symmetry directional WHIM index/weighted by atomic van der Waals volumes (WHIM descriptors)
18	1016	G1e	1^st^ component symmetry directional WHIM index/weighted by Sanderson electronegativities (WHIM descriptors)
19	1040	G3s	3^rd^ component symmetry directional WHIM index/weighted by atomic electropological states (WHIM descriptors)
20	1200	R8u+	R maximal autocorrelation of lag 8/unweighted (GETAWAY descriptors)
21	1295	nRCOSR	Number of thioesters (aliphatic) (Functional group counts)

Listed are the names, indices and a brief definition of the 21 descriptors selected as the optimized set in our angle distance model for odorant mixture similarity prediction.

### The model predicted similarity in separate datasets

**Figure 6:**
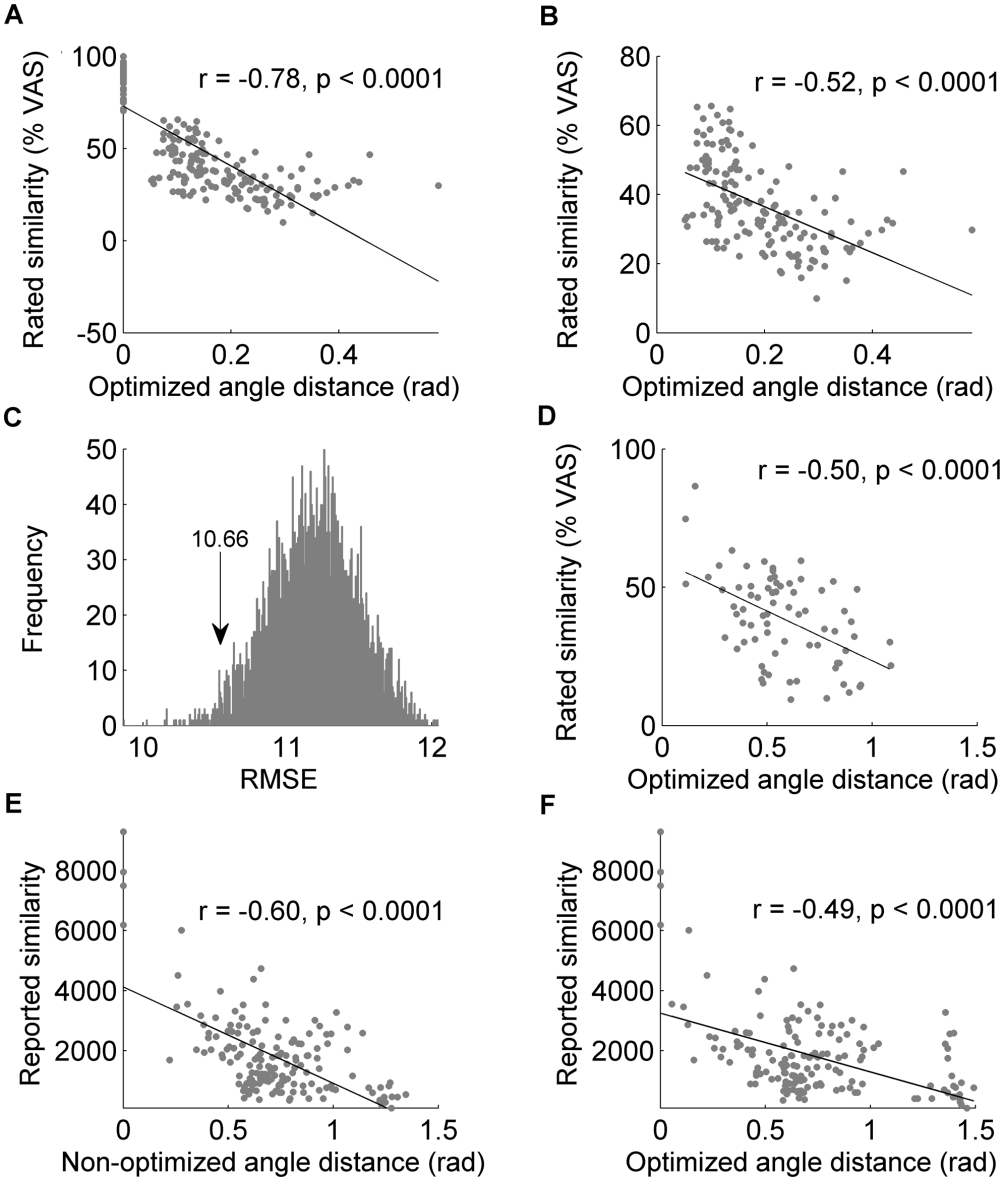
Performance of the optimized angle distance model on independent data. (**A**) Performance of the optimized model on complete Dataset #1 in Table S2. Each dot reflects a comparison between two mixtures. (**B**) The same as in panel A after omitting comparisons of mixtures to themselves. (**C**) RMSE histogram reflecting the performance of random selections of 21 descriptors. The optimized selection was at an RMSE of 10.66, which is better than 95.30% of the randomly selected sets. (**D**) Performance of the optimized angle distance model on mono-molecules (Dataset #3 in Table S2). (**E**) Performance of the angle distance model on mono-molecules tested 50 years ago independently by others [28]. (**F**) Performance of the optimized angle distance model on the data in panel E. Each dot reflects a comparison between two mono-molecules.

One might ask how well our model performs under different conditions. Recall that so far we had optimized our model on Dataset #2 in Table S2 consisting of mixtures ranging in size from 4 to 10 components. We now set out to test the performance of our model and selected descriptors on Dataset #1 in Table S2. This set not only includes larger mixtures but also includes 43 additional molecules not included in Experiment 2. Using this set we obtained a correlation of r = −0.78, p<0.0001 for all comparisons (Figure 6A), and r = −0.52, p<0.0001 for non-overlapping comparisons alone (Figure 6B). To further get a sense of how well this selection of descriptors performs on this data, we compared its performance to that of 4000 randomly selected sets of 21 descriptors. We measured the performance in terms of RMSE on Dataset #1 in Table S2. The selected set of 21 descriptors predicted similarity with an RMSE of 10.66. Compared to randomly selected sets of descriptors, the optimized set performed better than 95.30% of the sets (Figure 6C). Performance was tested using only the 147 comparisons between non-overlapping mixtures.

### The model predicted similarity in mono-molecules

One may ask how the model optimized and tested in odorant-mixtures performs with mono-molecules. To obtain similarity ratings for mono-molecules we pooled three experiments to form Dataset #3 in Table S2. The first experiment included similarity ratings by 21 subjects (11 female) between 14 pairs of mono-molecules; the second included similarity ratings by 17 subjects (9 female) between 20 pairs of mono-molecules, and the third included 19 subjects (6 female) rating 40 pairs of mono-molecules for similarity. In total, 49 mono-molecules were included in this experiment. The pool of molecules is included in the original pool of 86 molecules in Experiment #1 and includes 42 of the 43 in the pool of Experiment #2. In total, 74 comparisons were conducted amongst the 49 molecules. Out of these comparisons, 65% (48 comparisons) included at least one molecule that was not used in Experiment #2. Each comparison was repeated twice.

We applied our selected set of descriptors to Dataset #3 in Table S2. As before, we measured the RMSE of the prediction made based on the descriptors we selected. We obtained an RMSE of 13.825 and r = −0.5, p<0.0001 (Figure 6D). In comparison, using all descriptors gave r = −0.39, p<0.0001. Thus, the set of descriptors optimized on Dataset #2 in Table S2 improved the predictive performance of our model on Dataset #3 in Table S2. Notably, Dataset #3 in Table S2 consists of 7 additional molecules that were not included in Dataset #2 in Table S2 which was used to optimize the model. Moreover, as previously noted, 65% of these comparisons include at least one molecule that was not used in Experiment #2. This renders the test on Dataset #3 in Table S2 fairly unrelated to the set of molecules used to optimize the model.

### The model predicted similarity in mono-molecules studied independently

If our model is to be helpful to researchers in the field, it must be applicable to data collected by others. Most published studies on olfactory mixtures looked only at simple mixtures of 2 to 4 components, and moreover, most all did not post their raw similarity matrices. The lack of posted raw data holds true for most studies of mono-molecular perceptual similarity as well, with one notable exception that we are aware of: Wright and Michels (1964) [28] printed a large table containing the pairwise similarity ratings given by 84 subjects to a matrix of odorants that included 33 odorants not in our experiments or model building. We applied our model to their data. The angle-distance model, whether using the non-optimized or optimized descriptor set, yielded a significant correlation between predicted and actual pairwise odorant similarity (non-optimized: r = −0.60, p<0.0001 (Figure 6E); optimized: r = −0.49, p<0.0001 (Figure 6F); difference between r values: z = −1.34, p = 0.18). Thus, whereas Wright and Michels failed to predict perceptual similarity in their data [28], our model was a significant predictor of similarity in this data collected half a century ago. The statistically equal performance across the optimized and non-optimized descriptors when applied to this dataset may have resulted from several factors, including that the odorant selection criteria may have reflected the theory they were testing, that the molecules were not first diluted to equated intensity, and that these were indeed mono-molecules whereas our optimization was for the prediction of mixtures. However, the most likely explanation for this relates to their testing procedure: they compared similarity of all odorants to five anchor odorants. The five anchor odorants, by definition, are a skewed representation of olfactory space. Therefore, we take this as a reminder that researchers who set out to use the current model should consider both its optimized and non-optimized versions, especially in cases where the data may be skewed in olfactory space.

### Descriptors that predict neural activity were poorer predictors of perceptual similarity

Based on measures of neural activity and receptor responses, primarily in rodents, but also in humans, two independent studies obtained two alternative sets of optimal physicochemical odor descriptors [20], [23]. We set out to compare the performance of these sets of descriptors versus the current descriptors in predicting perceptual similarity. Application of the Haddad descriptor set (containing 32 descriptors) [20] and the Saito descriptor set (containing 20 descriptors) [23] to the testing set of Dataset #2 in Table S2 yielded RMSE = 12.4049, r = −0.3608, p = 0.01 and RMSE = 11.2255, r = −0.5364, p<0.0001, respectively. Although significant, these predictions are significantly weaker than those obtained with the optimized angle distance model (difference between r values, both z>3.16, both p<0.005).

## Discussion

In this manuscript we identify a model that allows predicting odorant-mixture perceptual similarity from odorant-mixture structure. In this, we take an initial modest step towards generating a measure for smell. The immediate impact of this result will be in the design of olfaction experiments probing both perception and neural activity, which can now be linked within a measurable predictive framework to the structure of odorant-mixtures. For example, one prediction of the model pertaining to mixtures that span olfactory space (e.g., Dataset #1 in Table S2), was that as the number of independent mono-molecular components in each of two mixtures increases, the two mixtures should gain in similarity, despite containing no components in common. In fact, the model predicted that at around 30 mono-molecular equally-spaced components, all mixtures should start smelling about the same (Text S1 Section 2, Figure S1). We recently verified this prediction, which culminated in the odor Olfactory White [29].

### Why the angle distance model

One may argue that there are countless potential paths to model the contribution of the various physicochemical descriptors to the perception of similarity, and therefore ask why angle distance model was selected. Here we will describe the evolution of this model in our efforts: The simplest and most naïve initial solution to the problem we addressed was the pairwise distance model, and our initial efforts centered on its optimization. Although the details of this effort are beyond the scope of a single manuscript, we will note that the main weakness of the pairwise distance model is, as previously noted, its implication that the more common molecules two mixtures share, the more different they will smell. This is not a problem in the lab, where one can select non-overlapping mixtures (e.g., Dataset #1 in Table S2). In the real world, however, many different mixtures will typically share many common components (e.g., Dataset #2 in Table S2). We initially tackled this by adding a parameter that assigned a variable weight to the distance between components of one mixture that were ‘close’ to components of the second mixture. We then added a second parameter that defined the threshold for being considered a ‘close’ point. We optimized the added parameters but the performance of the model did not improve and the inconsistencies remained. In an attempt to further generalize our model we tried replacing the Euclidean distance that defines the pairwise distance with other typical functions. Amongst the functions we tested was dot product. When we did so, the other parameters that were selected in the optimization process pointed to a unified weight for all components in the mixtures. That is equivalent to a dot product of the sum of vectors. That is, the data pointed to the dot product of sums of vectors as a good model. Once we were led to a dot product of a sum of vectors we also normalized by the size of the vectors to eliminate the effect of the sheer number of components in a mixture. At this point we were already very close to an angle distance metric, after all, the cosine of the angle is the normalized dot product. When we finally arrived at an angle distance model the results were consistent with the comparisons of identical mixtures and the correlation was much stronger even without any added parameters.

### Consistency with behavior and neurobiology

In simple terms, the superior performance of the angle-distance model over the pairwise-distance model suggests a system that does not consider each mixture component alone, but rather a system that, through some configurational process, represents the mixture as a whole. This is in fact highly consistent with olfactory behavior and neural representation. In behavior, humans are very poor at identifying components in a mixture, even when they are highly familiar with the components alone [30]. The typical maximum number of equated-intensity components humans can identify in a mixture is four, this number is independent of odorant type [31], and does not change even with explicit training [32]. Moreover, perceptual features associated with a mono-molecule will sometimes make their way into a mixture containing that molecule, but sometimes not, and the rules for this remain unknown [33]. In other words, like our algorithm, human perception groups many mono-molecular components into singular unified percepts. This pattern, referred to as either associative, synthetic, or configural, is in contrast to the alternative of retaining individual mixture component identity, referred to as dissociative, analytical, or elemental. Although these patterns are not mutually exclusive, evidence from perception points to a primarily configural process in olfaction. Mixture synthesis may begin with a balance of agonistic and antagonistic interactions between mono-molecules at olfactory receptors in the epithelium [34], [35] or at glomeruli in the olfactory bulb [36], [37]. Thus, when components compete for common receptors, they may be harder to pick out of the mixture [38]. The configural mechanisms in epithelium and bulb are further reflected in cortex where patterns of neural activity induced by a mixture are unique, and not a combination of neural activity induced by the mixtures' components alone [39]–[43]. In other words, like our algorithm, also at the neural level, the olfactory system treats odorant-mixtures as unitary synthetic objects, and not as an analytical combination of components [42]–[49].

### Limitations of the model

Although the model performed well, it has three notable limitations. The first is that the mixtures we studied were made of components that were first individually diluted to a point of equal perceived intensity. Intensity influences olfactory perception in complex ways [50]–[53], and some odorants, such as indole, can sharply shift in percept with changing intensity [54]. Moreover, whereas some odorants can increase the overall intensity of a mixture they are added to, other odorants can reduce overall mixture intensity [55]. Given this complexity, one may assume that when one of two mixtures under comparison contains intensity-sensitive molecules such as indole, the power of our model may diminish. Notably, the independent test of our model (Figure 6E, 6F) implied that perceived intensity equation may not be a condition for the model to apply in the case of mono-molecular odorants. That said, the model will likely break down in mixtures whose components were not at all equated for perceived intensity. With this in mind, future iterations of our model should try to incorporate recently developed models for the prediction of odorant detection threshold (as a proxy for intensity) [56]–[58]. These models may provide an *intensity coefficient* that would allow applying our model to mixtures made of components that were not first equated for intensity.

A second limitation is related to the odorants used for model building and testing. If the odorants represent only a limited portion of olfactory perceptual space, then our model may apply to this portion of olfactory space alone. To protect against this, we used the largest datasets we could find in order to build the model, and tested our model against subsets of the data not included in model building. Nevertheless, because the full extent of olfactory perceptual space remains poorly defined, this remains a potential limitation.

Finally, a similar limitation is in the selection of physicochemical features we modeled. Again, the more features one incorporates into a model, the smaller the risk of not capturing the relevant sources of variance, and we modeled more than a thousand features. That said, due to our dependence on such tools as Dragon software, we model a large set of structural features, but lack in physical features. Specifically, features such as boiling point, vapor pressure, diffusion, etc., which undoubtedly have a strong relation to olfactory perception, remain unrepresented.

In conclusion, despite the above-noted limitations, we provide an algorithm that allows predicting odorant-mixture perceptual similarity from odorant-mixture structure. The synthetic nature of the algorithm is consistent with the synthetic nature of olfactory perception and neural representation. This algorithm can now serve as a framework for theory-based selection of components for odorant-mixtures in studies of olfactory processing.

## Methods

### Subjects

We tested 139 normosmic and generally healthy subjects (63 women, between the ages of 21 and 45) who provided written informed consent to procedures approved by the Weizmann Institute Ethics Committee, and the Helsinki Committee.

### General procedures

The experiments were conducted in stainless-steel-coated rooms with HEPA and carbon filtration designed to minimize olfactory contamination. All interactions with subjects during experiments were by computer, and subjects provided their responses through a computer keyboard or mouse. Odorant mixtures were sniffed from jars marked arbitrarily, and presentation order was counterbalanced across subjects. In order to minimize olfactory adaptation, a ∼40 second inter-trial interval was maintained between presentations.

### Equated-intensity odorants

All odorants were purchased from Aldrich Chemicals (St. Louis, MO) in the highest available purity. All odorants were diluted with either mineral oil, 1,2-propanediol or deionized distilled water to a point of approximately equally perceived intensity. This perceived-intensity equation was conducted according to previously published methods [29]. In brief, we identified the odorant with lowest perceived intensity, and first diluted all others to equal perceived intensity as estimated by experienced lab members. Next, 24 naïve subjects (10 females) smelled the odorants, and rated their intensity. We then further diluted any odorant that was 2 or more standard deviations away from the mean intensity of the series, and repeated the process until we had no outliers. This process is suboptimal, but considering the natural variability in intensity perception, together with naïve subjects' bias to identify “a difference”, and the iterative nature of this procedure, any stricter criteria would generate an endless process.

### GCMS verification

To verify that our method of odorant-mixture preparation and delivery did not generate novel compounds, we submitted one set of mixtures (Dataset #2 in Table S2) to analysis with GCMS. In brief, we left the samples to sit in closed vials for several hours, then incubated over night at 50°C. This was done to accelerate the kinetics of any potential reactions that may have occurred. All the individual components (mono-molecules) of the mixtures were run separately, to ascertain their purity. The single peak retention times and corresponding spectrum identifications were noted and verified using Wiley Registry 9^th^ Edition/NIST 2008 combined mass spectral library (Wiley, New York, NY). The mixture samples were then subjected to the same GCMS method as the single components, and Total Ion Chromatogram peaks were validated to contain only the expected peaks of their constituting single components. Peaks with wide or abnormal shapes were subjected to further spectrum deconvolution using AMDIS software (NIST, Gaithersburg, MD), to assess potentially overlapping peaks. All analyses was made using an Agilent 7890 Gas Chromtograph coupled to Agilent 5975 Mass Spectrometer (Santa Clara, CA), integrated with a Gerstel headspace sampler (Mülheim an der Ruhr, Germany). Prior to injection samples were incubated in the Gerstel agitator for 5 minutes under 35°C and 250 rpm agitation. One ml of vial headspace gas was drawn into a heated syringe and injected to a split/splitless inlet that was kept at 250°C and a Split ratio of 5∶1. The GC method used a HP-5 MS column (30 m×0.25 mm×0.25 µm) and Helium as a carrier gas with 1.5 ml/min constant flow. Temperature program was 50°C for 3 minutes, 15°C/min ramp up to 250°C for 3 minutes. MS scans were conducted in Electron Impact mode (70 eV) from m/z 40 to 550, 2.86 scans/sec. MS source and Quad temperature were 230°C and 150°C, respectively.

### Pairwise similarity tests

In each trial, each subject was presented with two mixtures and was asked to rate their similarity on a VAS. The question at the top of the VAS was “To what extent are these two odors similar” (in Hebrew), and the VAS scale ranged from “not at all” to “highly”. In Dataset #1 the VAS was also numerated from 1 (“not at all”) to 9 (“very”), and in the remaining datasets it was not numerated. In both cases, the ratings were normalized within subjects to a scale of 0% to 100%. Each subject repeated the experiment on two different days to assess test-retest reliability. We applied an arbitrary cutoff whereby if the difference between 2 repetitions of the same comparison was greater than 70%, this rating was excluded. This amounted to 109 out of 2070 ratings (∼5%) in Dataset #1 in Table S2, and no deletions in Datasets #2 and #3 in Table S2. The ratings by subjects whose similarity ratings for identical mixtures were poorer by at least 2 standard deviations from the mean were discarded. This amounted to 3 subjects. The average rated similarities were calculated across subjects.

## Supporting Information

Figure S1 **Predicting olfactory white.** The mean angle between a theoretical mega-mixture made of 679 monomolecular components, and other non-overlapping mixtures made of increasing numbers of components (5000 randomly selected mixtures for each number of components from 2 to 80). Error bars are STD. In brown is the p value for a t-test between consecutive mixtures (running average of 5 comparisons), which is significant (Bonferroni corrected for 79 comparisons = 0.0006, red dashed line) constantly up to 25 vs. 26 components, yet only rarely beyond 36 components.
(TIF)

Table S1 **Odorants and their physicochemical descriptors.** The table contains 1433 descriptors for each of 1358 odorants we modeled. Odorants are identified by their CID code, and descriptors identified by their Dragon short name. Note that the values were truncated to the second decimal point in order to meet the PLoS Comp. Biol. supplementary file-size limitations. We did not check how such truncation influences the final results. Those interested in an non-truncated version can contact us directly.
(CSV)

Table S2 **Similarity ratings by dataset.** The table contains the average normalized similarity rating applied to each comparison, by dataset. Note that Dataset #1 is the same used in reference #29 of the manuscript, and Datasets #2 and #3 were collected for this study alone. The fourth list of CID numbers is from Wright and Michels (1964).
(PDF)

Text S1 **A text detailing an alternative deterministic selection of descriptors, and the computational steps to uncovering** ***olfactory white*** **.**
(PDF)
