# Supplementary figures and images for: Predicting Odor Perceptual Similarity from Odor Structure
Source: PLoS Comput Biol. 2013 Sep 12;9(9):e1003184. doi: 10.1371/journal.pcbi.1003184 (PMC3772038; doi:10.1371/journal.pcbi.1003184)

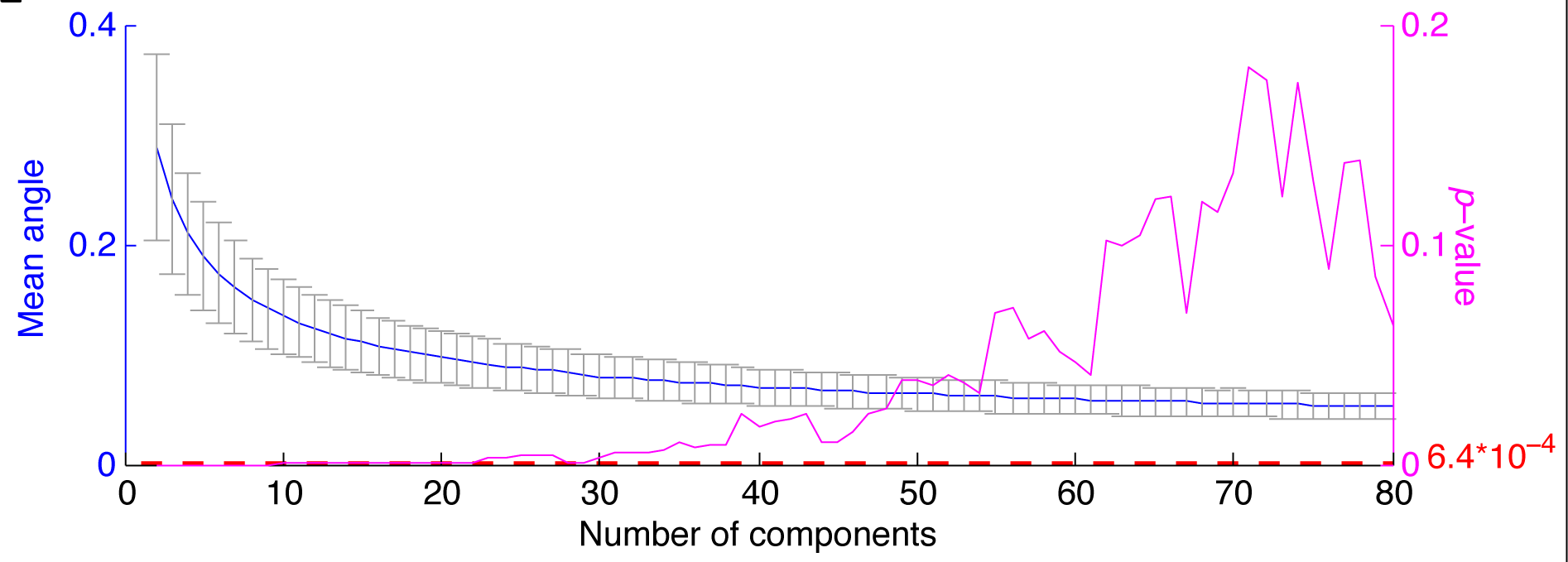

Supplement: Figure S1 — Predicting olfactory white. The mean angle between a theoretical mega-mixture made of 679 monomolecular components, and other non-overlapping mixtures made of increasing numbers of components (5000 randomly selected mixtures for each number of components from 2 to 80). Error bars are STD. In brown is the p value for a t-test between consecutive mixtures (running average of 5 comparisons), which is significant (Bonferroni corrected for 79 comparisons = 0.0006, red dashed line) constantly up to 25 vs. 26 components, yet only rarely beyond 36 components. (TIF) [file pcbi.1003184.s001.tif]
