# Supplementary material for: Predicting Odor Perceptual Similarity from Odor Structure
Source: PLoS Comput Biol. 2013 Sep 12;9(9):e1003184. doi: 10.1371/journal.pcbi.1003184 (PMC3772038; doi:10.1371/journal.pcbi.1003184)
Supplement: Table S2 — Similarity ratings by dataset. The table contains the average normalized similarity rating applied to each comparison, by dataset. Note that Dataset #1 is the same used in reference #29 of the manuscript, and Datasets #2 and #3 were collected for this study alone. The fourth list of CID numbers is from Wright and Michels (1964). (PDF) [file pcbi.1003184.s003.pdf]

Dataset #1 comparisons			
Comparison number	Mixture Number	Mixture Number	Average rated similarity
1	1	2	39.5833333333
2	1	3	34.8958333333
3	1	4	47.3958333333
4	1	5	49.4791666667
5	1	6	58.8541666667
6	1	7	43.75
7	8	2	24.4791666667
8	8	3	31.5104166667
9	8	4	15.1041666667
10	8	5	23.4375
11	8	6	19.2708333333
12	8	7	9.8958333333
13	9	2	43.2291666667
14	9	3	32.8125
15	9	4	57.5520833333
16	9	5	60.9375
17	9	6	55.2083333333
18	9	7	38.0208333333
19	10	2	43.2291666667
20	10	3	34.8958333333
21	10	4	45.8333333333
22	10	5	63.0208333333
23	10	6	58.8541666667
24	10	7	54.1666666667
25	11	2	48.9583333333
26	11	3	28.6458333333
27	11	4	53.125
28	11	5	65.625
29	11	6	61.9791666667
30	11	7	44.7916666667
31	12	2	22.9166666667
32	12	3	23.4375
33	12	4	30.2083333333
34	12	5	31.7708333333
35	12	6	36.9791666667
36	12	7	28.90625
37	13	14	24.5192307692
38	13	15	29.8076923077
39	13	16	29.3269230769
40	13	17	41.8269230769
41	13	18	43.2692307692
42	13	19	17.7884615385
43	20	14	28.8461538462
44	20	15	46.6346153846
45	20	16	24.5192307692
46	20	17	22.5961538462

47	20	18	27.8846153846
48	20	19	46.6346153846
49	21	14	26.4423076923
50	21	15	28.8461538462
51	21	16	42.7884615385
52	21	17	48.5576923077
53	21	18	46.6346153846
54	21	19	31.7307692308
55	22	14	26.4423076923
56	22	15	31.7307692308
57	22	16	54.8076923077
58	22	17	57.2115384615
59	22	18	50
60	22	19	20.6730769231
61	23	14	24.5192307692
62	23	15	32.6923076923
63	23	16	50
64	23	17	54.8076923077
65	23	18	58.1730769231
66	23	19	22.1153846154
67	24	14	22.1153846154
68	24	15	29.8076923077
69	24	16	26.4423076923
70	24	17	25.4807692308
71	24	18	22.1153846154
72	24	19	32.2115384615
73	25	26	28.8461538462
74	25	27	27.8846153846
75	25	28	37.0192307692
76	25	29	32.6923076923
77	25	30	33.6538461538
78	25	31	38.9423076923
79	32	26	18.75
80	32	27	27.8846153846
81	32	28	20.6730769231
82	32	29	38.9423076923
83	32	30	25.9615384615
84	32	31	24.5192307692
85	33	26	31.7307692308
86	33	27	38.4615384615
87	33	28	26.4423076923
88	33	29	46.6346153846
89	33	30	48.0769230769
90	33	31	27.4038461538
91	34	26	34.1346153846
92	34	27	36.5384615385
93	34	28	30.7692307692
94	34	29	47.5961538462
95	34	30	54.3269230769

96	34	31	30.7692307692
97	35	26	26.4423076923
98	35	27	34.6153846154
99	35	28	32.6923076923
100	35	29	37.0192307692
101	35	30	48.5576923077
102	35	31	34.6153846154
103	36	26	23.0769230769
104	36	27	34.6153846154
105	36	28	28.3653846154
106	36	29	19.2307692308
107	36	30	23.5576923077
108	36	31	17.3076923077
109	37	38	47.7272727273
110	37	39	37.5
111	37	40	35.7954545455
112	37	41	37.5
113	42	39	47.1590909091
114	42	40	46.0227272727
115	42	41	52.8409090909
116	3	38	22.1590909091
117	3	39	22.7272727273
118	3	40	27.2727272727
119	3	41	30.6818181818
120	43	44	34.0909090909
121	43	38	33.5227272727
122	43	45	15.9090909091
123	43	39	35.7954545455
124	43	40	34.6590909091
125	43	41	35.2272727273
126	43	46	31.25
127	47	44	33.5227272727
128	47	38	60.7954545455
129	47	45	21.0227272727
130	47	39	43.75
131	47	40	51.1363636364
132	47	41	46.5909090909
133	47	46	38.0681818182
134	48	44	32.3863636364
135	48	38	58.5227272727
136	48	45	24.4318181818
137	48	39	55.6818181818
138	48	40	65.3409090909
139	48	41	47.7272727273
140	48	46	47.1590909091
141	49	44	64.7727272727
142	49	38	54.5454545455
143	49	45	32.3863636364
144	49	39	38.0681818182

145	49	40	35.2272727273
146	49	41	40.9090909091
147	49	46	39.7727272727
148	8	8	95.3125
149	12	12	96.875
150	1	1	91.6666666667
151	9	9	91.6666666667
152	10	10	88.5416666667
153	11	11	85.4166666667
154	2	2	95.8333333333
155	3	3	95.8333333333
156	4	4	91.6666666667
157	5	5	87.5
158	6	6	95.8333333333
159	7	7	100
160	14	14	97.1153846154
161	15	15	93.2692307692
162	16	16	81.7307692308
163	17	17	87.5
164	18	18	87.5
165	19	19	81.7307692308
166	13	13	91.3461538462
167	20	20	91.3461538462
168	21	21	92.3076923077
169	22	22	91.3461538462
170	23	23	88.4615384615
171	24	24	94.2307692308
172	32	32	100
173	36	36	100
174	25	25	90.3846153846
175	33	33	94.2307692308
176	34	34	95.1923076923
177	35	35	82.6923076923
178	27	27	87.5
179	31	31	75
180	26	26	76.9230769231
181	26	26	90.3846153846
182	29	29	89.4230769231
183	30	30	90.3846153846
184	38	38	89.7727272727
185	39	39	71.5909090909
186	40	40	81.8181818182
187	41	41	86.3636363636
188	42	42	86.3636363636
189	43	43	76.1363636364
190	47	47	70.4545454545
191	48	48	79.5454545455

Dataset #1

Mixture number	Mixture Cids
1	[6501 264 2879 7685 7731 326 7888 61138 8030 1183]
2	[240 93009 323 8148 7762 3314 460 6184 798 6054]
3	[7710]
4	[31276 93009 11002 323 7966 8148 7632 22201 19310 7762 2758 3314 460 443158 20859 7059 999 6544 7770 10430]
5	[10890 93009 11002 6982 323 8797 7966 8148 7632 31252 19310 7762 3314 460 6184 8892 8103 12178 5281168 798 443158 20859 7059 91497 999 10821 6544 7770 7714 10430]
6	[7710 31276 10890 240 93009 11002 6982 323 8797 7966 8148 24915 7632 22201 31252 19310 7762 26331 2758 3314 460 8130 6184 8892 8103 12178 5281168 798 443158 20859 7059 62444 91497 999 10821 6054 6544 7770 7714 10430]
7	[93009 460 443158 6544]
8	[5283349]
9	[7410 6501 264 5281515 6259976 307 7685 326 5283349 7749 7363 7888 7119 8635 8918 6736 8030 5634 7921 1183]
10	[7410 6501 7600 7519 264 5281515 6259976 307 2879 7685 7731 326 5283349 7583 7749 7363 8129 7888 61016 8635 8918 957 7991 61138 6654 8118 6736 10722 1140 1183]
11	7991 61138 6654 8118 6736 8030 6989 10722 1140 5634 7921 1183]
12	[7731 7749 7888 1183]
13	[22201 7749 460 61016 7119 61138 999 10821 6054 6544]
14	[323 7762 7363 7888 16666 8635 7059 7991 6736 8030]
15	[7059]
16	[10890 7519 323 7583 7762 26331 8892 7888 443158 16666 8635 91497 8918 957 18827 8118 8030 6989 5634 10430]
17	[14286 31276 7600 7519 11002 6982 307 323 5283349 7762 26331 3314 7363 8892 8103 7888 443158 16666 8635 7059 91497 957 18827 7770 6736 8030 6989 10722 5634 10430]
18	[14286 7710 31276 10890 7600 7519 11002 6982 307 323 2879 7731 5283349 7583 7762 26331 3314 7363 8892 8103 7888 443158 16666 8635 7059 62444 91497 8918 957 18827 7991 7770 8118 6736 8030 6989 10722 5634 10430 7921]
19	[7731 8892 7888 7059]
20	[62336]
21	[6501 264 6259976 8797 7685 7632 22201 2758 460 8129 5281168 62336 798 61016 20859 61138 10821 6054 1140 1183]
22	[6501 62433 264 5281515 7685 326 7966 8148 24915 7632 22201 31252 19310 2758 8130 8129 6184 12178 798 61016 7119 20859 999 10821 6054 6544 6654 1140 7714 1183]
23	[7410 6501 62433 240 93009 264 5281515 6259976 8797 7685 326 7966 8148 24915 7632 22201 31252 19310 7749 2758 460 8130 8129 6184 12178 5281168 62336 798 61016 7119 20859 61138 999 10821 6054 6544 6654 1140 7714 1183]
24	[7749 61138 6054 6544]

25	[7600 62433 307 5283349 443158 8635 8918 999 6736 10722]
26	[7410 10890 7519 7685 24915 26331 8129 16666 7770 10430]
27	[7714]
28	[7410 10890 93009 6259976 2879 8797 7685 24915 8103 5281168 7888 16666 18827 7991 6054 6654 7770 8030 5634 10430]
29	[7410 10890 93009 11002 8797 7685 7731 7966 24915 7583 26331 5281168 7888 798 61016 16666 7119 20859 18827 7991 6054 6654 7770 8118 8030 1140 7714 5634 10430 1183]
30	[7410 10890 7519 93009 11002 6259976 323 2879 8797 7685 7731 326 7966 24915 31252 7583 26331 460 8129 8103 5281168 7888 798 61016 16666 7119 20859 18827 7991 10821 6054 6654 7770 8118 8030 1140 7714 5634 10430 1183]
31	[5281168 10890 2879 7966]
32	[31276]
33	[14286 7710 31276 7600 5281515 6982 5283349 7632 7762 3314 6184 443158 91497 8918 957 61138 999 6544 6989 7921]
34	[14286 7710 31276 7600 62433 240 264 5281515 6982 307 5283349 7632 22201 19310 7749 2758 3314 6184 8892 443158 7059 91497 8918 957 61138 6544 6736 6989 10722 7921]
35	[14286 6501 7710 31276 7600 62433 240 264 5281515 6982 307 5283349 8148 7632 22201 19310 7762 7749 2758 3314 7363 8130 6184 8892 12178 62336 443158 8635 7059 62444 91497 8918 957 61138 999 6544 6736 6989 10722 7921]
36	[62433 7363 443158 61138]
37	[14286 7600 3314 16666 91497 18827 7991 7770 6989 5634]
38	[61199 10890 93009 264 6259976 24915 7762 460 8129 5281168 443158 8918 957 999 17100]
39	[61199 6501 264 2879 7731 326 24915 7762 460 8129 8892 12178 5281168 443158 4133 999 10821 17100 11552 1183]
40	[61199 6501 31276 10890 7519 240 93009 11002 6259976 2879 7685 7731 326 5283349 7583 460 8129 8892 12178 443158 4133 8918 957 61138 999 10821 6544 17100 11552 1183]
41	[61199 6501 31276 10890 7519 240 93009 264 11002 6259976 2879 8797 7685 7731 326 7966 5283349 9609 24915 7583 7762 26331 460 8129 8892 12178 5281168 443158 20859 4133 62444 8918 957 61138 999 10821 6544 6736 17100 31277 10722 11552 1183]
42	[7410 7710 7600 62433 307 7749 3314 61016 16666 91497 6054 6654 8030 5634 10430]
43	[7410 14286 7710 7600 62433 5281515 6982 22201 7749 3314 8130 8103 7888 16666 91497 6654 7770 5634 10430 7921]

44	[6501 8797 326 26331 8129 12178 999 6544 6736 11552]
45	[7685]
46	[6501 460 999 6544]
47	[7410 7710 7600 62433 5281515 6982 307 7632 22201 31252 19310 7749 3314 6184 7888 61016 16666 7119 91497 18827 7991 6054 6654 7770 8118 8030 6989 7714 5634 10430]
48	[7410 14286 7710 7600 62433 5281515 6982 307 323 8148 7632 22201 31252 19310 7749 2758 3314 7363 8130 6184 8103 62336 7888 798 61016 16666 7119 8635 7059 91497 18827 7991 6054 6654 7770 8118 8030 6989 1140 7714 5634 10430 7921]
49	[7600 62433 7991 6989]

Dataset #2 comparisons			
Comparison number	Mixture number	Mixture number	Average rated similarity
1	1	2	42.8920768277
2	1	3	38.2925188853
3	1	4	58.2205435883
4	5	6	29.7321081182
5	5	7	62.231981175
6	5	3	59.6834225837
7	2	5	56.8320625991
8	2	6	31.1102534239
9	8	2	45.1906525188
10	8	9	55.8460436439
11	6	7	27.1032905381
12	6	10	28.4666081119
13	6	11	37.8212120261
14	12	5	29.2264463261
15	12	2	32.8488419076
16	12	6	35.9348363339
17	12	10	37.0957060269
18	7	1	35.8676065026
19	7	8	38.8315476659
20	3	12	29.3431840677
21	3	13	41.8740722418
22	3	4	55.1835934311
23	3	10	44.6881379562
24	9	5	61.8433647714
25	9	12	30.0817078966
26	9	3	49.1864076834
27	9	14	54.434006142
28	13	1	45.0479865702
29	13	2	43.3056175159
30	13	6	40.0733972789
31	4	5	71.0763747141
32	4	8	51.6250918479
33	4	13	37.7755842727
34	4	11	42.65543746
35	10	9	51.6787465177
36	10	4	60.041397948
37	14	1	34.334684991
38	14	6	33.6834812847
39	14	7	66.8014539949
40	14	13	40.4904882931
41	14	10	65.2906207311
42	11	1	62.0149033493
43	11	2	52.1849505052
44	11	8	48.0076235013
45	11	12	34.7939733695
46	11	13	50.4446400068

47	1	5	63.4176348598
48	1	8	35.9579997488
49	1	6	44.5168647674
50	1	12	53.8750343555
51	1	9	46.8743338229
52	1	10	37.0116310677
53	5	8	47.6427082577
54	5	13	37.6277234001
55	5	10	47.5206029328
56	5	14	56.5273711569
57	5	11	55.5547834727
58	2	7	56.5124839064
59	2	3	47.8892521298
60	2	9	56.4702011828
61	2	4	61.0520828953
62	2	10	59.0501557976
63	2	14	64.6282394837
64	8	6	30.0333647715
65	8	12	24.9943769886
66	8	3	50.605626467
67	8	13	23.3561339388
68	8	10	46.2247464518
69	8	14	38.2099169932
70	6	3	35.1094674536
71	6	9	27.793943301
72	6	4	28.1503345953
73	12	7	33.8501517588
74	12	13	36.6066038191
75	12	4	27.5310341851
76	12	14	39.1216385083
77	7	3	53.5510491156
78	7	9	58.2561770446
79	7	13	43.9005771667
80	7	4	61.4611468128
81	7	10	50.0969153042
82	7	11	65.5970916721
83	3	14	48.393467523
84	3	11	50.3668346769
85	9	13	41.7041072969
86	9	4	58.5990436446
87	9	11	69.0992488397
88	13	10	39.051042677
89	4	14	63.0563164143
90	10	11	45.9789168529
91	14	11	62.5123193783
92	1	1	70.2901207791
93	5	5	58.2890207475
94	11	11	69.6266983069
95	14	14	68.4690574039

Dataset #2

Mixture number	Mixture Cids
1	[326 26331 6544 1140]
2	[7710 62433 7519 7685 3314]
3	[31276 62433 7519 8129 12178 18827 10722]
4	[62433 8797 2758 3314 8635 61138 6054 6544 10722]
5	[7410 240 93009 8635]
6	[7519 8148 31252 8103 5281168 6544]
7	[240 307 7731 2758 12178 62336 8635]
8	[31276 8148 7762 18827 7714]
9	[7710 93009 8130 8103 5281168 7059 8918 7714]
10	[11002 307 7685 12178 4133 7991 6054 7770 7714]
11	[240 2758 8130 8129 5281168 7059 4133 8918 957 6654]
12	[7410 326 2758 62444 7770 1140]
13	[7410 7519 11002 8797 8129 5281168 6654 8030]
14	[8797 7731 7966 3314 62336 7059 7991 61138 6054 6544]

**Dataset #3 comparisons**			
Comparison number	CID	CID	Average rated similarity
1	7410	19310	14.6836842105
2	7710	7749	30.4985
3	31276	3314	42.0935
4	7519	8129	48.2145
5	240	8103	59.6205
6	93009	12178	48.0875
7	11002	62336	34.136
8	7685	8635	51.213
9	7731	62444	11.8755
10	326	8918	53.1495
11	8148	7991	49.2995
12	9609	61138	52.0752631579
13	22201	1140	15.3265
14	31252	10430	27.067
15	31276	26331	21.4161181775
16	6054	31276	47.2128292008
17	240	326	40.274739359
18	93009	240	52.9339534823
19	7685	7762	33.6845511624
20	8148	93009	13.9985159777
21	7762	8129	39.8168634983
22	7749	7519	63.3664229027
23	26331	8148	41.4196129139
24	3314	11002	20.7514735389
25	62336	22201	15.9907826016
26	7059	7685	59.2873712665
27	4133	31252	32.2346601009
28	8030	62336	30.1873834883
29	7519	8030	34.9673839804
30	326	7059	50.4071198269
31	22201	7714	15.6632745813
32	31252	6054	37.5950057271
33	8129	4133	48.3947495328
34	6654	7714	31.1489998864
35	7410	3314	40.218635755
36	7410	12178	53.7903638806
37	7710	307	49.0692920675
38	7710	8130	31.8349010241
39	61138	7410	18.3113202284
40	10821	7710	41.5299748449
41	6544	31276	37.1413111883
42	8797	8130	49.9228372081
43	7731	8797	51.5322298853
44	8103	8148	22.6170029804
45	12178	62433	43.1011214638
46	5281168	2758	36.8672525169

47	5281168	8103	56.9387376195
48	62444	240	9.8495700282
49	957	8148	22.6042669165
50	957	8129	86.6011726073
51	18827	93009	30.1616686586
52	7991	2758	16.6954798309
53	10821	7731	51.3415861985
54	6544	5281168	53.6374800249
55	7770	307	42.8196854198
56	8118	240	29.1133737842
57	8118	11002	74.7306409532
58	62433	957	57.6383061725
59	62433	8030	21.7308202652
60	10722	7731	55.9731866722
61	1140	26331	29.1266581311
62	307	7991	9.3496150027
63	8797	10821	48.8879859743
64	2758	10722	57.8195838198
65	8129	6054	46.3080427806
66	8129	7770	40.35004591
67	8103	8918	49.638579144
68	12178	7714	36.3096194455
69	62444	1140	14.8323293991
70	8918	7059	50.351401804
71	8918	61138	27.6837704748
72	7991	10722	19.1925053919
73	61138	6054	26.0335811646
74	7770	6544	44.4479882844

Wright & Michels - Dataset CID's
CID
7888
17100
637566
8842
8184
8174
8914
263
1031
702
5943
638011
22311
6448
241
8078
9253
8079
8882
180
1254
637511
1032
176
996
2969
264
16590
402
6736
1049
7222
7969
