# Supplementary material for: Predicting Odor Perceptual Similarity from Odor Structure
Source: PLoS Comput Biol. 2013 Sep 12;9(9):e1003184. doi: 10.1371/journal.pcbi.1003184 (PMC3772038; doi:10.1371/journal.pcbi.1003184)
Supplement: Text S1 — A text detailing an alternative deterministic selection of descriptors, and the computational steps to uncovering olfactory white . (PDF) [file pcbi.1003184.s004.pdf]

Supplementary text for:

Predicting Odor Perceptual Similarity from Odor Structure

Kobi Snitz[1*], Adi Yablonka[1*], Tali Weiss[1], Idan Frumin[1], Rehan M Khan, Noam Sobel[1]

[1]Department of Neurobiology, Weizmann Institute of Science, Rehovot 76100 Israel

*These authors contributed equally

Correspondence to:

kobi.snitz@weizmann.ac.il

Department of Neurobiology, Arison Building,

Weizmann Institute of Science, Rehovot 76100 Israel

Tel: (+) 972 8 934 6253

1. Selecting Chemical descriptors using Minimum Redundancy Maximum Relevance Feature Selection (mRMR)

The manuscript-described selection of an optimized subset of descriptors involves random selections and may give rise to different descriptor subsets in recurring simulations. We thus set out to repeat the descriptor subset selection process using a different, deterministic method. To do so, we used a method that considers minimal mutual information between descriptors and the measure to be evaluated, i.e. rated similarity [28]. This method uses a measure of mutual information to select the relevant features without redundancy. It uses information about the category of the observation to carry out the calculation. That is, in our case this method uses information about the average rated similarity to select chemical descriptors relevant to it. The data for the program is a matrix of observations and a list of categories for each of the observations. In our case the categories were the average rated similarities between mixtures and the data matrix described the comparisons between the mixtures. We used the mutual information distance script mrmr_mid_d [28] to select the best 25 descriptors based on the data matrix representing the comparisons in the training set. We tested the performance of this selection on the testing set of comparisons in Dataset #2 as we did for the other method. The results were RMSE = 11.5888 and $r = -0.4908$, $p < 0.005$. This result was significantly poorer than that obtained with the optimized descriptor set (Fisher's r-to-z transformation, $z = 3.42$, $p < 0.0006$). It should be noted that although the mRMR method uses information about the rated similarity to select descriptors it does not actually consider the measurement of prediction as we do in the simulation method.

2. Predicting an olfactory white

A prediction of the angle-distance model is the existence of a point, in terms of number of components, where all mixtures will tend to smell similar, a point we will call olfactory white. According to our model, this point corresponds to the percept generated by a mixture having the mean values of each of the physicochemical features. To simulate this point, we calculated the coordinates of a mega-mixture containing 679 odorants, namely half of our available database. Next we calculated the predicted perceptual similarity between this mixture and increasingly large mixtures, each randomly selected 5000 times from the second half of the database, i.e., the mixtures under comparison shared no components in common. We observed that the angle distance between the megamixture and mixtures of increasing size leveled off from as early as ~30 components (Supplementary Figure 1). To further estimate the point of leveling, we conducted t-tests on the predicted angle between the megamixture and consecutive odorant mixture sizes. The first point at which angles for consecutive mixture sizes were not significantly different was at 25 components, and from 36 components and more, consecutive mixtures were only rarely significantly different (Supplementary Figure 1). We conclude with a conservative estimate that predicted similarity began to level off at 30 ± 10 components. This suggests that any mixture of 30 ± 10 components will be perceptually similar to any other non-overlapping mixture of 30 ± 10 components, or phrased differently, a 30 ± 10 point random sample is a sufficiently good estimator of the mean. These predictions, of course, assume that the components are well distributed in the physicochemical space, and are of equal perceived intensity.
